# Parental regret regarding children’s vaccines—The correlation between anticipated regret, altruism, coping strategies and attitudes toward vaccines

**DOI:** 10.1186/s13584-016-0116-1

**Published:** 2016-11-07

**Authors:** Yaira Hamama-Raz, Eyal Ginossar-David, Menachem Ben-Ezra

**Affiliations:** School of Social Work, Ariel University, Ariel, Israel

**Keywords:** Attitudes toward vaccines, Altruism, Coping strategies, Regret, Anticipated regret

## Abstract

**Background:**

Parental hesitancy for recommended childhood vaccines is a growing public health concern influenced by various factors. This study aimed to explore regret regarding parental decisions to vaccinate their children via possible correlations between anticipated regret, altruism, coping strategies, and parents’ attitudes toward the vaccination of their children.

**Methods:**

The study was conducted during 2014 in Israel. Data were collected via snowballing methodology (i.e., Internet forums, Facebook and e- mails). 314 parents of children ages 0–6 years participated in the study. Questionnaires were distributed and completed on-line including attitudes toward vaccines, altruism, coping strategies, regret and anticipated regret.

**Results:**

Pearson analysis revealed a moderate negative association between attitudes toward vaccinations and regret. In addition, weak but significant positive associations emerged between anticipated regret and regret as well as between gender and regret.

Performing hierarchical regression analysis revealed contribution of 35.9 % to the explained variance of regret suggesting that coping strategy of instrumental support, attitudes toward vaccinations and anticipated regret are linked significantly to regret.

**Conclusion:**

Parental attitudes toward vaccines and anticipated regret have a salient role when deciding whether or not to vaccinate children and contribute to the prediction of regret regarding vaccination. In order to increase parental consent to vaccination of their children, it is important to minimize possible regret through the strength of the recommendation and/or knowledge base about risk/benefit (perceived, heuristic) of vaccines that might influence parental attitudes and lessen their anticipated regret.

**Trial registration:**

N/A. This is not a clinical trial and thus does not require registration. Ethics approval was received from Ariel University School of Social Work Ethics committee (18/02/14). This was an attitude survey.

The Ariel University School of Social Work Ethics committee approved performance of this attitude survey (18/02/14).

## Background

Immunization has proven successful for controlling and eliminating life-threatening infectious diseases and preventing an estimated two to three million deaths each year [1]. However, ensuring widespread vaccination coverage is complicated by a wide range of factors, including vaccine hesitancy, which leads to uncertainty in segments of the public about the safety and efficacy of vaccinations. Actually, decision-making regarding vaccination is a complex process which is dependent on emotional, cultural, social, spiritual and political factors as well as on cognitive factors [2]. According to the World Health organization (WHO), vaccine hesitancy refers to a delay in acceptance or refusal of vaccines despite availability of vaccination services [3].

With regard to vaccine hesitant parents, studies have revealed that although parental vaccine concerns vary according to knowledge and personal experience, the underlying premise remains remarkably constant: fear that vaccines (and/or their additives) are unsafe, will transmit to the immunized person the infection against which they are designed to protect, or that somehow getting the “natural” disease is healthier [4]. Actually, for many parents, immunization remains an emotional issue since choosing to vaccinate is an action that involves a deliberate intervention involving the child. In contrast, having a child fall prey to an illness that could have been prevented results from an omission, the failure to vaccinate. Action or inaction toward vaccination might result in feelings of regret, which is the aversive interplay of feelings resulting from the comparison of a selected choice and a non-selected alternative [5]. In light of this, Kumar et al. [2] claimed that the causes of vaccine hesitancy can be described as a complex interaction of external, agent-specific and host-specific factors such as immunization requirements, policies, media, norms, vaccine-efficacy, vaccine safety, race, education, income and knowledge about vaccines. However, despite their valuable contribution to the understanding of vaccine hesitancy, psychological factors have not received much attention in their review. Likewise, Valen [6] focused on vaccine hesitancy in a local, culture-dependent setting in the Israeli population and did not relate to specific psychological factors. Nevertheless, he acknowledged that in Israel vaccine hesitancy appears to be a process where individuals exercise self-determination and self-empowerment and make their own decisions based on assessment, reflection, choosing between various options and dealing with considerable complexities. Since sense of regret might be part of this process, the aim of the current study was to explore personal variables that might play a role in predicting regret of parental decision making toward childhood vaccination among Israelis parents.

In Israel, immunization of children is not required by law however the Israel Ministry of Health strongly recommends a vaccination program for babies and children [7]. According to the Health ministry, about 95 % of babies and children are taken to well-baby clinics for their routine vaccinations, These include shots against tetanus, diphtheria and whooping cough plus hemophilus influenza B and polio (combined quintuple vaccine); measles, mumps and German measles plus chicken pox (combined quadruple vaccine); Prevnar against pneumococcus; hepatitis B; hepatitis A [7]. Babies receive their first vaccines at well baby clinics operated by the Ministry of Health (i.e., Tipat Halav). Children and adolescents continue to receive vaccinations in line with the Ministry of Health schedule throughout elementary and high school. Since it is the parents’ responsibility to bring children up to age six to well-baby clinics to receive vaccinations, we decided to focus on parents of children age 0–6 years.

A possible theory that might contribute to understanding the decision-making process regarding administration of vaccines is Rosenstock’s Health Belief Model (HBM) [8]. The HBM hypothesizes that health-related action depends upon the simultaneous occurrence of three classes of factors: (1) The existence of sufficient motivation (or health concern) to make health issues salient or relevant. (2) The belief that one is susceptible (vulnerable) to a serious health problem or to the sequelae of that illness or condition. This is often termed perceived threat. (3) The belief that complying with a particular health recommendation would be beneficial in reducing the perceived threat, and at a subjectively acceptable cost. Cost refers to perceived barriers that must be overcome in order to follow the health recommendation, including but not restricted to financial expense.

Inspired by the HBM theory we proposed anticipated regret, altruism, attitudes toward vaccines and coping strategies as variables that might predict regret regarding the parent’s decision to vaccinate their children. In line with the idea that people might take into account emotional reactions to possible outcomes when making decisions, Janis and Mann [9] suggested focusing more on the psychological aspects of anticipated regret. According to Janis and Mann, “anticipatory regret is a convenient generic term to refer to the main psychological effects of the various worries that beset a decision maker before any losses actually materialize…Such worries, which include anticipatory guilt and shame, provoke hesitation and doubt, making salient the realization that even the most attractive of the available choices might turn out badly” ([9], p. 222).

It has been proposed that anticipated regret is a better predictor than cognitive risk estimates in predicting vaccination uptake [10–13]. In a recent study [14] regret of anticipated inaction was found to be a factor that mediated the relationship between risk perceptions and vaccination intent among a sample of American parents with young children.

Altruism was another variable that was proposed as a predictor of regret regarding the decision to vaccinate. Altruism is a psychological trait that can influence human behavior and decision-making thereby influencing the outcomes of vaccination strategies and other public health policies [15]. It can be considered a state of personal agency in which regard for another is the primary motivation, despite relatively little expectation of foreseen reciprocity or personal gain [16]. Likewise, Shim et al., [17] noted that altruism significantly shifted vaccination decisions away from individual self-interest and towards the community optimum, greatly reducing the total cost, morbidity and mortality for the community. As such, they stated that promoting altruism could be a potential strategy to improve public health outcomes. However, research investigating the role of altruism in vaccination is scant, and most studies revolved around the altruistic nature of participating in vaccine trials and health promotion research [18–22]. Moreover, in a systematic review Quadri-Sheriff et al., [23] revealed that there appears to be some parental willingness to immunize children for the benefit of others (“herd immunity”), but its relative importance, as a motivator is largely unknown. Further work is needed to explore this concept as a possible motivational tool for increasing childhood immunization.

Another variable that might link to regret concerning decision making regarding vaccination is coping strategies. Coping strategies refer to the specific efforts, both behavioral and cognitive, that people use to master, tolerate, reduce or minimize stressful events [24]. Two major categories of coping strategies are widely recognized: problem‐solving strategies such as efforts to do something to actively alleviate stressful circumstances, and emotion‐focused coping strategies, such as efforts to regulate the emotional consequences of stressful or potentially stressful events [24, 25]. With respect to vaccinations, very few studies related to how individual differences in coping strategies may influence health behavior, including the decision to be vaccinated [26, 27], however they deal with adult decision making for themselves and not for their children. Hence, in the current study we choose to relate to two coping strategies—emotional support (related to emotion‐focused coping strategies) and instrumental support (related to problem‐focused coping strategies). It has been generally accepted that social support is a proactive coping strategy that helps to mediate stress [28, 29].

To summarize, the purpose of the current study was to explore the correlation between altruism, coping strategies (i.e., emotional and instrumental support), parental attitudes toward vaccination and anticipated regret to regret regarding parents’ decisions to vaccinate their young children. We hypothesized that: 1). Parents who exhibit high emotional and instrumental support of coping strategies would reveal lower regret toward their decision to vaccinate their young children in comparison to those who engage with low emotional and instrumental support of coping strategies 2) Parents with higher levels of altruism would exhibit less regret toward their decision to vaccinate their young children in comparison to parents with low levels of altruism; 3) Parents with positive attitudes toward vaccines would report less regret toward their decision to vaccinate their young children in comparison to parents with negative attitudes toward vaccines; 4) Parents with higher levels of anticipated regret will reveal higher regret in comparison to parents with low levels of anticipated regret.

## Methods

### Sample and procedure

The study was conducted in Israel during the year 2014,, following the summer of 2013 when the Israeli government launched a nationwide vaccination campaign, in an attempt to inoculate all children under age ten with oral polio vaccine (OPV), a form of the vaccine containing a live, weakened form of the virus [30]. Upon receiving approval from the Institutional Review Boards of the School of Social Work at the university to which the authors are affiliated, data were collected via snowballing methodology. Three hundred fourteen participants completed an internet survey. The authors posted messages in Hebrew asking parents of young children (up to age six) to participate in an online survey, which was advertised through various means such as Israeli parenting forums (e.g., Tapuz forums for parents of babies/toddlers/children in elementary school) social-network sites (e.g. Facebook) and e-mails to colleagues, neighbors and friends that further distributed the messages to their friends and acquaintances and so on. Clicking on the hypertext link led to a dedicated site that included the study questionnaire. Each participant was required to click on the informed consent tab in order to participate in the study. IP addresses were obtained for each participant in order to prevent duplications and the possibility that one participant would fill out questionnaires in both groups. The IP address was deleted after quality control was performed, in order protect to participant anonymity.

### Instruments

Participants completed the following battery of self-report questionnaires:

- *Demographic data* questionnaire that included age, marital status, number of children, children’s ages, education, place of residence, participants’ health status and degree of religiosity.
- *Medical data regarding vaccines* were collected from responses to the following questions: Did you vaccinate your children in the past? (Yes/No); How many of your children were vaccinated?; In the list of vaccines, please indicate which vaccinations your children received; Do you intend to vaccinate your children in the future? (Yes/No/Not Sure). The list relates to: vaccine against Diphtheria-Tetanus-Whooping cough + Haemophilus influenzae type B + Polio [DTaP-Hib-IPV]; Vaccine against Measles-Mumps-Rubella (German measles) + Varicella (chicken pox) [MMRV];Vaccine against pneumococcus bacteria [PCV]; Vaccine against Hepatitis B; Vaccine against Hepatitis A.
- *Attitudes toward vaccination* were examined using the Attitudes toward Vaccination Scale [31]. This self-report questionnaire examines attitudes toward vaccines in general, utilization and safety. It contains 11 items asking respondents to indicate how much they agree with each statement on a 3-point Likert scale (0 = strongly disagree, 1 = unsure; 2 = strongly agree) which represented a progressively more positive attitude toward vaccination. The responses were then summed to arrive at a total score ranging from 0 (most negative attitude toward vaccines) to 22 (most positive attitude toward vaccines). Reliability of the original scale yielded high internal consistency (α = 0.88) as well as for the current study (α = .84).
- *Altruism* was assessed using the self-report Altruism Scale [32] that asked respondents to report the frequency with which they had engaged in 20 everyday behaviors such as “I have given directions to a stranger” and “I have given money to a charity”. Participants reported the frequency of their altruistic behavior on a five-point scale (1 = never, 2 = once, 3 = more than once, 4 = often and 5 = very often). Possible scores ranged from 20 to 100, higher scores indicated higher altruistic tendencies. The scale demonstrated high internal consistency (α = 0.78–0.87 for the original scale and α = 0.80 for the current study).
- *Coping Strategies*—Emotional and instrumental support coping strategies were measured using the short version of the COPE scale [25]. Respondents were asked to indicate the extent to which each of the two strategies was used in coping with everyday problems. Responses were rated on a four-point scale, ranging from 0 = Not at all to 3 = Great extent; and transformed into a 1–4 scale. Each subscale was composed of two items.
- *Regret* toward the decision to vaccinate was examined using the Decision Regret Scale (DRS) [33], which assessed the decision maker’s regret associated with a healthcare decision. The DRS is a 5-item scale that asks subjects to reflect on a particular decision and then rate each item on a 5 point scale from 1 (strongly agree) to 5 (strongly disagree) (e.g., “I would decide the same thing If I had to decide again). A total DRS score is derived from reverse scoring items 2 and 4, calculating a mean score, and transforming the mean scores by subtracting 1 and then multiplying by 25. The total DRS scores range from 0 to 100 and higher scores indicate a greater appraisal of decision regret. The scale showed good internal consistency (α = 0.81 to 0.92 in the original scale and in the current research α = 0.86).
- *Anticipated regret* toward the decision to vaccinate was examined with a single item- “Do you agree with the following statement: anticipated regret is one of my considerations when I decide to vaccinate my children” on a 6 point scale (1 = completely disagree to 6 = completely agree). It is based on previous studies that used a single item to measure anticipated regret of decisions [11, 13, 33, 34].

### Statistical procedure

Data were analyzed using SPSS version 21. All variables were summarized using standard descriptive statistics such as frequency, means and SD. Pearson’s correlation were performed to examine associations between socio-demographic and independent variables and regret. Independent sample *t*-tests were conducted to examine differences among groups across socio-demographic and independent variables related to regret. Hierarchical regression analyses were carried out to assess the contribution of socio-demographic, attitudes toward vaccines, coping strategies, altruism, anticipated regret and interactions between these variables to the variance in the parents’ regret. Additionally, we examined potential multicollinearity between the study variables, and found no indication of multicollinearity, as tolerance levels ranged from 0.704 to 0.944 and variation inflation factor levels ranged from 1.059 to 1.421, which is in line with literature requirements [35].

## Results

### Participant characteristics

Of the 314 participants, 82.2 % were women, average age was 36.17 (SD = 7.2), most were married (89.8 %). Parents to one or two children comprised 74.2 % of the sample (the average age of children was 2.06; SD = 1.05). Most of the participants were secular (74.8 %) and reported excellent or good health. The average years of education was 16.67 SD = 2.7. Comparison of the characteristics of the study population to those of the general population (as can be seen in Table 1), revealed differences: The study group included more women than men, more married couples, and most of the sample belong to the age group of 29–38 while in the general population 49+ is the largest age group. The average number of years of education of our sample was higher for both men (17.3 vs. 13.9) and women (16.8 vs. 14.2). These differences might stem from the recruiting procedure employed in the study- a snowballing sample of participants who completed an internet survey and who are parents to children up to age six (potential biases are discussed in the limitation section).

**Table 1:** Characteristics of the Study Sample (*N* = 314) and those of the general Israeli population

Variables		Sample (%)	^a^Israeli Population (%)
Age	18–28	8.0	22.0
	29–38	60.2	20.0
	39–48	28.3	16.9
	49+	3.5	40.1
Sex	Men	10.2	48.8
	Women	89.2	51.2
Marital status	Single	3.5	30.7
	Married	89.2	55.2
	Divorced/Separated	7.3	8.6
Education	Men	17.3	13.9
	Women	16.8	14.2

^a^According the Israeli Central Bureau of Statistics (CBS) (http://www.cbs.gov.il)

With regard to medical data, 100 % reported that they vaccinated their children against Diphtheria-Tetanus-Whooping cough + Haemophilus influenzae type B + Polio; 100 % against Measles-Mumps-Rubella (German measles) + Varicella (chicken pox) ;32.2 % against pneumococcus bacteria [PCV]; 81.5 % against Hepatitis B; 77.4 % against Hepatitis A. Beyond that, 43 % of the participants had no regrets at all and 57 % of the participants showed regret on a spectrum ranging from little regret to substantial regret. Anticipated regret was distributed according to the following categories: Completely Disagree 28.2 %, Mostly Disagree 20.2 %, Slightly Disagree 9.3 %, Slightly Agree 18.6 %, Mostly Agree 16.0 %, and Completely Agree 7.7 %.

### Correlations between the study’s variables and regret

Pearson analysis was performed to evaluate the associations between coping strategies of emotional and instrumental support, altruism, attitudes toward vaccinations, anticipated regret and regret, as shown in Table 2.

**Table 2:** Pearson correlations between research variables

	Descriptive Statistics	1	2	3	4	5	6	7	8	9	10
1. Age	Mean = 36.17 (SD 7.25)		-.279*^**^	.391^***^	.151^*^	-.143^*^	-.114	.058	-.014	.003	-.029
2. Gender	N = 282 (89.8 %)			-.141^*^	-.053	.234^***^	.131^*^	.012	-.022	.138^*^	.120^*^
3. Number of children	Mean = 2.06 (SD = 1.05)				-.012	-.192^***^	-.176^**^	.097	-.113^*^	.081	.014
4. Years of education	Mean = 16.67 (SD = 2.66)					.119^*^	.057	-.019	.135^*^	-.068	-.072
5. Emotional support	Mean = 6.63 (SD = 1.49)						.475^***^	-.011	.156^**^	.056	-.041
6. Instrumental support	Mean = 6.54 (SD = 1.23)							.095	.112^*^	.042	-.111
7. Altruism	Mean = 3.07 (SD = 0.47)								-.099	.132^*^	.076
8. ATV^a^	Mean = 2.68 (SD = 0.37)									-.168^**^	-.511^***^
9. Anticipated regret	Mean = 2.97 (SD = 1.69)										.263^***^
10. Regret	Mean = 2.97 (SD = 1.69)										

^a^Attitudes towards vaccinations

***p < 0.001

**p < 0.01

*p < 0.05

A moderate negative association was found only between attitudes toward vaccinations and regret (*r* = −.511, *p* < 0.001), that is, those who had favorable attitudes toward vaccinations reported less regret toward their decision to vaccinate their children. A weak but significant positive association emerged between anticipated regret and regret (*r* = .263, *p* < 0.001) suggesting that parents who felt higher rates of anticipated regret toward vaccinating their children, revealed higher rates of regret. In addition, a very weak positive association emerged between gender and regret (*r* = .120, *p* < 0.05), suggesting that mothers revealed more regret toward their decision to vaccinate their children in comparison to fathers.

### The contribution of the independent variables to the explanation of variance in regret

Hierarchical regression analyses were performed to ascertain the unique and cumulative contributions of the independent variables to the explanation of the variance in regret. Demographic variables (age, sex, number of children, years of education) were entered in the first step. Emotional and instrumental support coping strategies, altruism, attitudes toward vaccines and anticipated regret entered in the second step. Before performing the hierarchical regression analysis, the multicollinearity assumption was rejected, with the maximal VIF measure of predictors being 1.4.

Table 3 presents the regression coefficients for the explanation of the variance in regret.

**Table 3:** Factors associated with Regret (*n* = 314)

Variables	Statistics			
	B	S.E	t	*P* value
Regret				
Step 1				
Age (years)	.001	.007	.044	.965
Sex (women vs. men)	.249	.150	1.657	.099
Number of Children (high vs. low)	-.116	.085	−1.368	.172
Years of education (high vs. low)	.004	.045	.098	.922
Step 2				
Age (years)	.001	.006	.045	.964
Sex (Women vs. Men)	.115	.127	.909	.364
Number of Children (high vs. low)	-.099	.070	−1.417	.158
Years of education (high vs. low)	-.051	.038	−1.354	.177
Emotional support (high vs. low)	.018	.028	.628	.530
Instrumental support (high vs. low)	-.073	.034	−2.181	.030
Altruism (high vs. low)	-.014	.081	-.171	.865
Attitudes towards vaccination (favorable vs. unfavorable)	−1.054	.114	−9.259	<.001
Anticipated regret (high vs. low).	.085	.022	3.829	<.001
Model Summary				
Step 1	*R* = .140; *R* ^2^ = .020; *R* ^2^ change = .020; *F* (4249) = 1.247; *p* = .292			
Step 2	*R* = .599; *R* ^2^ = .359; R^2^ change = .340; *F* (5244) = 25.858; *p* < .001			

As can be seen in Table 2, the independent variables contributed 35.9 % to the explained variance of regret (Step 1 contributed 2 % to the explained variance of regret while step 2 contributed 34 %). Specifically, the coping strategy of instrumental support was linked significantly and negatively to regret (β = −.073, *p* = .030), suggesting that parents who utilized instrumental support exhibited lower regret toward the decision to vaccinate. In addition, attitudes toward vaccinations was correlated significantly and negatively to regret (β = −1.054 *p* < .001). That is, parents with favorable attitudes toward vaccination revealed lower regret. Finally, anticipated regret was linked significantly and positively to regret (β = .85, *p* < .001). That is, parents who reported anticipated regret toward vaccinating the child exhibited higher regret regarding the decision to vaccinate their children.

## Discussion

In the present study, we examined the relationship between coping strategies (instrumental and emotional support), altruism, parental attitudes toward vaccines and anticipated regret, to regret toward the decision to vaccinate the child. The main findings of the study indicate that those with more favorable attitudes toward vaccines, those who use the coping strategy of instrumental support and those who report lower anticipated regret revealed less regret toward the decision to vaccinate their children.

Specifically, with respect to attitudes toward vaccines, our findings show that this factor played a key role in parents’ regret toward vaccinating their children. At first glance, this finding might appear to be trivial as one might claim that if the attitudes toward vaccination are positive, it is self-evident that regrets after vaccinating are low.

However, favorable attitudes toward vaccines do not always link to low regret; sometimes regret will be greatest when the outcome of action leads to a worse outcome than the outcome of inaction (for example vaccination that did not result in the anticipated protection) [36].

A possible explanation for our result regarding the link between positive attitudes and regret may stem from the Health Belief Model [8]. According to this model, when one considers a health preventive action, such as vaccination, four factors are taken into account: perceived susceptibility, severity, benefits, and barriers. In addition to these factors symbols for action are added (for example- broadcasts for immunization) and influencing variables such as personality, socioeconomic status, knowledge, gender and age [37]. Thus, parents with positive attitudes towards vaccines would think about their child’s illness (perceived susceptibility), the level of the risk of illness (severity), the decreased risk of illness infection(benefits) and the contribution of the vaccine to the child’s health (in this case- the lack of barriers). Thus, regret toward vaccines might be infrequent. In other words, using positive frames that focus on the gains of a preventative treatment may be most persuasive and may lessen a possible regret toward vaccination action. Studies have found past behaviour [13, 16, 17] and knowledge [18, 19] to be further predictors of intention and behaviour of action to vaccinate.

Another explanation may be attributed to the role of physicians, which was found to be the most important source of information regarding vaccination [38, 39]. Thus, we may assume that when parents trust in their physicians they will have positive attitudes toward children’s vaccinations and will therefore exhibit less regret regarding the vaccination of their children [40].

With respect to coping strategies, our findings show that instrumental support as a coping strategy related to lower levels of regret toward vaccines while emotional support was not found to be linked to regret. A possible explanation may be attributed to the content of each coping strategy; seeking social support for instrumental reasons is seeking advice, assistance, or information. This is a problem-focused coping strategy. However, seeking social support for emotional reasons is getting moral support, sympathy, or understanding. This is an aspect of emotion-focused coping strategy [25]. Since vaccine hesitancy has been found to be influenced by factors such as complacency, convenience and confidence [41], it seems that information and professional advice are more essential in reducing regret than sympathy or sharing. In line with this notion, previous research has indicated that propagation of “fear stories” likely has contributed to the growth of vaccine hesitancy in the US and internationally [42, 43].

Regarding anticipated regret which was found to link to regret toward the decision to vaccinate the child, our result was in line with previous research concerning the role of anticipated regret in accepting seasonal influenza [44]. A possible explanation may be related to Zeelenberg & Pieters’ [45] explanation that ‘Regret is experienced when people realize or imagine that their present situation would have been better had they decided differently in the past” (p. 214). Thus, experienced regret may ultimately fuel anticipated regret, potentially allowing people to learn from their regret experiences [46]. This mutual connection might explain our result. In addition, it has been shown that anticipated regret plays a more important role than risk perception [47]. For example, Christy et al. [48] found among unvaccinated undergraduate men, that anticipatory emotions (i.e. anticipated regret) played a more central role in decision-making regarding HPV vaccination than cognition related to vaccination.

Finally, with respect to altruism, our findings showed no link to regret. These findings were not previously reported. A possible explanation might be related to the concept of “Self-Protective Altruism” noted by Hirschberger [49]. According to this concept in cases where there is an arousal of thoughts of death, orientation to altruism decreases. Therefore, people who have an altruistic tendency in most situations in their lives may abandon this tendency when death anxiety arises (whether consciously or not). Another explanation might be related to the fact that the study was conducted in a population that is characterized by a high commitment to childhood vaccination [8]. Even the emergency polio vaccination campaign in the summer of 2013 indicated that the compliance rates with polio vaccination were 82 % among “immediate deciders” and 70 % among “late deciders” [6]. Thus, it could be that altruism in such a compliant population became less relevant in predicting regret toward vaccination of children.

### Limitations

In sum, our findings highlight the salience of parents’ attitudes toward immunization in predicting regret. The level of regret after vaccination of a child, depends on the parent’s position regarding the vaccines. Nevertheless, this study had several limitations. First, there is greater representation of women than men. It was noted previously that women are more likely to answer internet questionnaires than men are [50, 51], and that mothers are the ones who make most health decisions for the family including decisions regarding vaccinations [52], therefore, caution should be taken in generalizing the findings. Likewise, our participants were found to have higher levels of education in comparison to the general Israeli population, suggesting a possibility for a bias in the findings, which may limit the representativeness of the findings for the population as a whole. Second, the current study did not focus on specific vaccines for children, and was concerned with vaccines in general from birth to age six. Therefore, we cannot rule out that each participant referred to a different type of vaccine in response to the questions, suggesting that future studies should refer to a specific vaccine. Third, the sampling of participants in the current study was done through snowball- online questionnaires that were transferred to a potential group of participants that further distributed them to their friends and acquaintances and so on. This sampling method could represent only a specific population segment and is not necessarily representative of the general population of parents in Israel. Moreover, the questionnaire was transferred online. Alongside the benefits of online questionnaires such as comfort, speed and convenience sampling analysis, there are several disadvantages such as obtaining data from those who tend to respond to internet questionnaires due to their accessibility and availability on the internet, impersonal delivery, unclear instructions that cannot be discussed, concern of respondent anonymity [53, 54].

### Implications

The findings also have important implications. Medical staffs including social workers and psychologists who deal with public health should be encouraged to develop psychosocial interventions that can supply information and knowledge regarding immunization by setting up psycho-educational groups for parents about the benefits of childhood vaccinations, prevalence of immunization, importance, and side effects. In addition, these interventions may include enhancing of coping strategies that will reduce potential regret and may contribute to the process of decision making. From the policymakers’ perspective, it seems that targeting feelings of anticipated regret through eliciting thoughts of potential negative outcomes or imagining experiencing a negative feeling may also lead to more positive actions and can also be a persuasive mechanism. Accordingly, parents may benefit from the recognition and normalization of any regret anticipated during their discussions with medical professionals in the decision making process as previous evidence indicated that nurses and physicians were found to have an important effect on parents’ opinions and are considered role knowledgeable [26, 27].

## Conclusions

The current research contributes to our understanding of how a sense of regret might be part of vaccine hesitancy suggesting that personal variables such as positive attitudes toward vaccines, using the coping strategy of instrumental support and lower anticipated regret predicted regret of parental decision making toward childhood vaccination among Israeli parents. This approach of integrating attitudes to vaccination, regret, anticipated regret, coping strategies and altruism can perhaps be applied to examine vaccination programs less consensual than routine childhood vaccination. For example, HPV vaccination among adolescents [48] or flu vaccination [44].

Further research is suggested to examine this option, alongside including pairs of parents regarding our research question. In addition, since in the present study altruism was not found to be a predictive variable of regret, it is possible that examining personality traits of the “five factors model of personality” such as agreeableness which includes pro-social elements may yield different results.

## Abbreviations

DRS: Decision Regret Scale
HBM: Health Belief Model
IP: Internet protocol address
OPV: Oral polio vaccine
PCV: Pneumococcus bacteria
SD: Standard deviation
WHO: World Health Organization
